# Incidence and Predictors of Cognitive Frailty Among Older Adults: A Community-based Longitudinal Study

**DOI:** 10.3390/ijerph17051547

**Published:** 2020-02-28

**Authors:** Nurul Fatin Malek Rivan, Suzana Shahar, Nor Fadilah Rajab, Devinder Kaur Ajit Singh, Normah Che Din, Hazlina Mahadzir, Noor Ibrahim Mohamed Sakian, Wan Syafira Ishak, Mohd Harimi Abd. Rahman, Zainora Mohammed, Yee Xing You

**Affiliations:** 1 Nutritional Sciences Programme and Centre for Healthy Ageing and Wellness (H-CARE), Faculty of Health Sciences, Universiti Kebangsaan Malaysia, Jalan Raja Muda Abdul Aziz, Kuala Lumpur 50300, Malaysia; fatinmalek93@gmail.com; 2 Dietetics Programme and Centre for Healthy Ageing and Wellness (H-CARE), Faculty of Health Sciences, Universiti Kebangsaan Malaysia, Jalan Raja Muda Abdul Aziz, Kuala Lumpur 50300, Malaysia; yeexing@gmail.com; 3 Biomedical Science Programme, Centre for Healthy Ageing and Wellness (H-CARE), Faculty of Health Sciences, Universiti Kebangsaan Malaysia, Jalan Raja Muda Abdul Aziz, Kuala Lumpur 50300, Malaysia; nfadilah@ukm.edu.my; 4 Physiotherapy Programme & Centre for Healthy Ageing and Wellness (H-CARE), Universiti Kebangsaan Malaysia, Jalan Raja Muda Abdul Aziz, Kuala Lumpur 50300, Malaysia; devinder@ukm.edu.my; 5 Health Psychology Programme and Centre of Rehabilitation Science, Faculty of Health Sciences, Universiti Kebangsaan Malaysia, Jalan Raja Muda Abdul Aziz, Kuala Lumpur 50300, Malaysia; normahcd@ukm.edu.my; 6 Internal Medicine & Geriatric Department, Pusat Perubatan Universiti Kebangsaan Malaysia, Jalan Yaacob Latif, Bandar Tun Razak, 56000 Batu 9 Cheras, Kuala Lumpur 50300, Malaysia; drhazlina2013@gmail.com; 7 Occupational Therapy Programme, Centre for Healthy Ageing and Wellness (H-CARE), Faculty of Health Sciences, Universiti Kebangsaan Malaysia, Jalan Raja Muda Abdul Aziz, Kuala Lumpur 50300, Malaysia; nooribrahim@ukm.edu.my; 8 Audiology Programme, Centre for Healthy Ageing and Wellness (H-CARE), Faculty of Health Sciences, Universiti Kebangsaan Malaysia, Jalan Raja Muda Abdul Aziz, Kuala Lumpur 50300, Malaysia; wsyafira@ukm.edu.my; 9 Optometry and Vision Sciences Programme, Centre for Healthy Ageing and Wellness (H-CARE), Faculty of Health Sciences, Universiti Kebangsaan Malaysia, Jalan Raja Muda Abdul Aziz, Kuala Lumpur 50300, Malaysia; harimirahman@ukm.edu.my (M.H.A.R.); zainora@ukm.edu.my (Z.M.)

**Keywords:** cognitive frailty, frailty, mild cognitive impairment, incidence, predictors, aging, depression

## Abstract

(1) Background: Cognitive frailty (CF) is the simultaneous presence of physical frailty and cognitive impairment with an increased risk of dementia. Considering that the risk factors of CF are mostly elucidated from cross-sectional studies, we conducted a community-based longitudinal study to determine the incidence and the predictors of CF among Malaysian older adults.; (2) Methods: Out of 490 older adults participating in the Malaysian Towards Useful Aging (TUA) study, 282 were successfully followed-up at five-years for an analysis of the CF incidence. CF was defined as a comorbid physical frailty (>1 Fried criteria) and mild cognitive impairment (Petersen criteria). A comprehensive interview-based questionnaire was administered for sociodemographic information, cognitive function, physical function, dietary intake, psychosocial, and biochemical indices. Univariate analyses were performed for each variable, followed by a regression analysis to identify the predictors of CF that accounted for confounding effects between the studied factors; (3) Results: The incidence rate of CF was 7.1 per 100 person-years. Advancing age (OR=1.12, 95% CI:1.04-1.21, p < 0.05), depression (OR=1.20, 95% CI:1.05-1.37, p < 0.05), decreased processing speed, assessed by a lower digit symbol score (OR=0.67, 95%CI:0.0.56-0.80, p < 0.05), decreased functional mobility measured using Timed-Up-and-Go (TUG) (OR=1.23, 95% CI:1.04-1.46, p < 0.05), low vitamin D intake (OR:0.36, 95% CI:0.14-0.93, p < 0.05) and physical frailty (OR=2.16, 95% CI:1.02-4.58, p < 0.05) were predictors for CF incidence; and (4) Conclusions: Our study results could be used as an initial reference for future studies to formulate effective preventive management and intervention strategies to decelerate CF development among older adults.

## 1. Introduction

Dementia is one of the most common disabling diseases among older persons. It is believed that the underlying pathologies of dementia are present for many years before cognitive loss becomes apparent. Early treatments for dementia [1,2] have led to a search for the prodromal stage of the disease, such as cognitive frailty (CF), that could predict its future progression. CF is a clinical construct introduced by the International Academy on Nutrition and Aging (I.A.N.A) and the International Association of Gerontology and Geriatrics (I.A.G.G), defined in 2013 as a heterogeneous clinical manifestation characterized by the comorbidity of physical frailty operationalized with the Cardiovascular Health Study (CHS) phenotypic model and cognitive impairment diagnosed with a Clinical Dementia Rating (CDR) scale of 0.5 among older adults without a concurrent diagnosis of Alzheimer’s diseases (AD) or other dementias [3]. CF was designated with the goal of identifying individuals with reduced cognitive reserve that is a potentially reversible consequence of frailty rather than the result of neurodegenerative disorders [3]. CF is reported to increase the incidence of dementia [4], and it is associated with adverse health outcomes, such as functional decline, disability, poor quality of life [5], and increase in mortality [6]. This calls for the urgent formulation of a preventive action plan against the development of CF among older adults. 

CF has been consistently shown to increase the risk of the progression toward dementia among older adults [4,7]. It was highlighted in the Singapore Longitudinal Ageing Studies (SLAS) that the risk of neurocognitive disorders increased three times among older adults with the co-existence of physical frailty and cognitive impairment, as compared to those with a single syndrome [8]. It is noteworthy that physical and cognitive decline shared a common underlying mechanism, as both these conditions involved the same areas of brain regions and networks [9,10]. The upstream causative mechanisms of physical frailty include systemic inflammation, cell senescence, hormonal changes, and mitochondrial dysfunction [11,12]. These mechanisms are reported to exacerbate neurodegenerative and vascular damages associated with cognitive decline and dementia [13,14]. 

Sarcopenia, characterized by muscle dysfunction, slow gait velocity, and cognitive decline is also known to increase the risk of developing dementia [15]. Sarcopenia is potentially associated with the CF phenotype, as this condition increases the risks of both physical frailty and cognitive impairment [16]. Even though sarcopenia and physical frailty are often used interchangeably, these two phenotypes have different constructs, whereby sarcopenia indicates a progressive decline in the lean body mass or muscle mass that contributes to frailty development [16,17]. Hence, the link between physical and cognitive impairments as relevant predictors of dementia warrants the need for a multidimensional approach in the assessments and interventions of older adults with CF. 

The global prevalence of CF was reported to be between 1.0%–9.8%, respectively [5,8,18,19]. However, the incidence rate of CF is yet to be reported. It is important to note that prevalence is just a snapshot of a figure for a condition at a particular point in time, whereas incidence measures the rate of occurrence of new cases for a particular condition among a specified population in a given period of time [20]. Hence, a prospective investigation to determine the magnitude of CF as indicated by the incidence rate and the predictors of CF among multi-ethnic Malaysian older adults is essential to formulate public health strategies for a healthy longevity. In this cohort study, we aim to determine the incidence and possible predictors of CF through a five-years follow-up among multi-ethnic older adults in Malaysia.

## 2. Materials and Methods 

**Figure 1:**
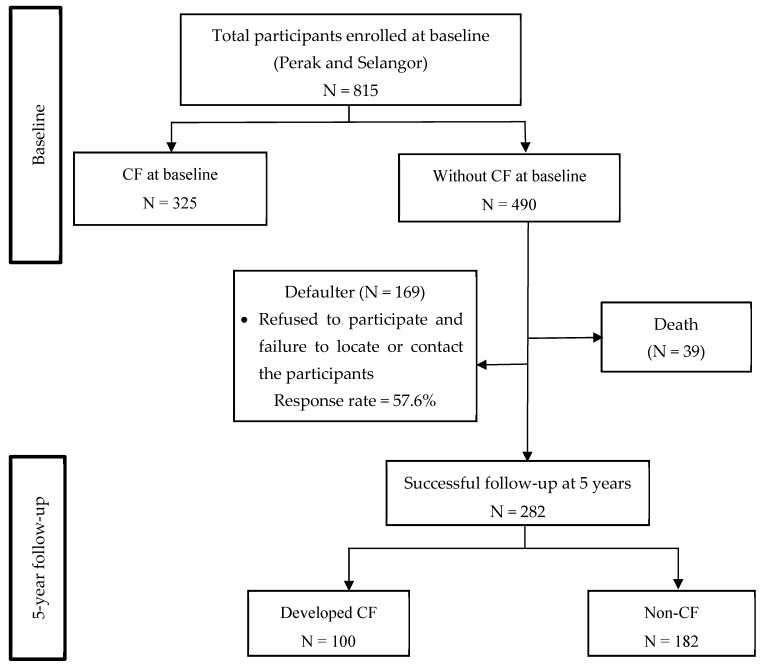
Illustration of the number of participants from the baseline to the 5-year follow-up for CFNotes: CF = cognitive frailty.

This is a follow-up study of the Longitudinal Study on Neuroprotective Model for Healthy Longevity (LRGS TUA) cohort [21] at the 5 years endpoint. At the baseline, a total of 815 older adults consisting of 372 men and 443 women were recruited through a multi-stage random sampling procedure from two states, namely Selangor and Perak (representing central and northern regions of Malaysia), as reported earlier [18]. A total of 325 participants were categorized into the CF group at the baseline, while another 490 participants were grouped in non-CF (Figure 1). It is noteworthy that only non-CF participants were included in the follow-up assessments for the incidence analysis. After 5 years, 39 participants had died, while 169 participants refused to participate and failed to be located or contacted. Hence, a total of 282 non-CF participants at the baseline were successfully followed up at 5 years. Different fieldworkers were recruited for the follow-up assessments to comply with the blinded assessors criteria. This study was approved by the Medical Research and Ethics Committee of the Universiti Kebangsaan Malaysia (UKM1.21.3/244/NN-2018-145). A written information was given, and informed written consent was acquired from all participants prior to participation. 

### 3.1. Data Collection

Participants were interviewed by trained enumerators using a standardized questionnaire, and measurements for a number of parameters were performed at their respective community centers. The questionnaire consists of information on the socio-demography, health status, neuropsychological and psychosocial functions, lifestyle, dietary intake, blood pressure, anthropometry, physical fitness, and functional status, as reported by Shahar et al. [21]. Measurements were performed at the baseline and during the 5 years follow-up period. The descriptions of the measuring instruments are provided below. The participants were allowed to rest in between the tests, and monetary incentives were provided for the participants who completed the assessment. 

### 3.2. Incidence and Operational Definition of Cognitive Frailty

The incidence of CF is referred to the development of either or both physical pre-frailty/frailty and mild cognitive impairment (MCI) during the 5 years follow-up among the participants who did not exhibit CF at the baseline. A participant was characterized as CF if they satisfied the operationalization definition of CF as proposed in our previous report [18]. Participants were categorized into two final groups: CF and non-CF groups. 

Frailty was assessed using Fried et al. [22] criteria, a well-known frailty assessment used in the Cardiovascular Health Study. Frailty consisted of five components in this assessment: weight loss, exhaustion, low physical activity, weakness, and slowness. The presence of one or two of these criteria was categorized as pre-frailty and three or more criteria as frailty. As proposed previously, participants with pre-frailty and frailty were grouped together as one target group [18]. The operationalized measurements used to define frailty criteria were as following:

1. Shrinking, subjective report of unintentional loss of weight around 5 kg in the prior year (i.e., not due to diet or exercise).
2. Self-report of exhaustion and poor endurance and energy, defined by using two items of the Center for Epidemiologic Studies Depression scale (CES-D): (a) I felt that everything I did was an effort; and (b) I could not get going; (the question was how often in the last week did you feel like this (rare = 0, some or little time (1–2 days) = 1, moderate (3–4 days) = 2, and most of the times = 3). Participants who scored two and above were categorised as frail based on this exhaustion criteria.
3. For the low physical activity, a low physical activity was assessed using the Physical Activity Scale for Elderly (PASE), and a low physical activity was identified by low scores (in the lowest tertile) of the PASE score.
4. Slowness was defined using the five-meter gait speed test with the cut-off points stated in the original reference adjusted for the gender and height.
5. The last criterion was weakness. It was defined using a hand grip strength test with the cut-off points stated in the original reference adjusted for the gender and body mass index.

Participants with mild cognitive impairment (MCI) were classified according to the criteria set by Petersen et al. [23] and Lee et al. [24], which included subjective memory complaints by participants or caregivers, as indicated by the answer ‘Yes’ in the question ‘Do you have memory complaints?’, objective memory impairment (at least 1.5 SD below the mean for either RAVLT or digit span test), no or very minimal functional limitations in basic activities of daily living (ADL), a preserved global cognitive function as assessed using a M-MMSE score of ≥19, and reporting no evidence of dementia as reported by an authorized doctor at the baseline [25]. Participants without all of these criteria were considered cognitively normal. 

### 3.3. Possible Predictors of Cognitive Frailty:

Variables reported previously [14] were examined, including:

#### 3.3.1. Socio-demographic Data and Health Condition

The socio-demographic and health variables obtained included gender, ethnicity, age, living arrangement, marital status, educational level (number of years in formal education), and household income. The data on cigarette smoking and alcohol drinking were also collected at the baseline. With regard to the smoking status, participants were asked: “Have you ever smoked a cigarette?”. The following criteria were used to define the smoking status: never-smokers (fewer than 100 cigarettes in their lifetime), former smokers (more than 100 cigarettes in their lifetime, but had not smoked in the past 30 days), and current smokers (more than 100 cigarettes in their lifetime and had smoked in the past 30 days) [26]. In order to obtain data on alcoholic drinking, participants were asked: “Have you ever drunk alcoholic beverages?”. Furthermore, the participants were classified as follows: never-drinkers (never consumed alcoholic beverages in their lifetime), former drinkers (consumed at least once in their lifetime), and current drinkers (had consumed alcohol beverages for the past 12 months) [27]. Besides this, self-reported information on certain diseases (diabetes mellitus, hypertension, hypercholesterolemia, and heart diseases) diagnosed by doctors in the prior years were recorded. In epidemiology studies, self-report of disease diagnoses has been used widely and reported to be valid [28]. The living arrangement was described as the condition of either staying with others or living alone. The mortality data (death) was obtained from the Communicable Disease Control Unit of the Ministry of Health Malaysia. This unit compiled nationwide data, including death (obtained from the national registry center), for the purpose of epidemiological analyses.

#### 3.3.2. Nutritional Status, Body Composition, and Blood Pressure

Nutritional status indicators were assessed using anthropometric measurements, including weight, height, arm span, waist circumference, hip circumference, mid-upper arm circumference (MUAC), and calf circumference. The measurement of these figures was done according to standard protocols [14]. The Body Mass Index (BMI, kg/m^2^) was calculated as the body weight in kilograms divided by the squared standing height in meters. The body circumferences were measured using a non-extensible and flexible plastic measuring tape. The body composition was assessed via Bio-electrical Impedance Analysis (BIA) Inbody S10 (Bio-space Co. Ltd, Seoul, Korea). The systolic and diastolic blood pressure was also measured using a calibrated digital automatic blood pressure monitor (OMRON, Kyoto, Japan).

#### 3.3.3. Cognitive Function Test 

The global cognitive function was measured using the Malay Version Mini-Mental State Examination (MMSE) and the Malay version of Montreal Cognitive Assessment (MoCA) [29]; the attention and working memory were evaluated using the Weschler Memory Scale-Revised (WMS-R) and the Digit Span Forward and Backward [30]; information processing was assessed using the Digit Symbol test and the Rey Auditory Verbal Learning Test (RAVLT) for verbal learning and memory [31]. 

#### 3.3.4. Physical Fitness and Functional Status

Physical fitness was assessed using the Senior fitness test (SFT) [32]. A total of six fitness tests were conducted, namely 2-minute step (endurance), back scratch (upper body flexibility), chair sit and reach (lower body flexibility), chair stand (lower body strength), timed up and go (TUG) (balance and mobility), handgrip strength (upper body strength), and rapid pace gait speed tests. The activity of Daily Living (ADL) [33] and Instrumental Activity of Daily Living (IADL) [34] was also administered to assess the functional status. 

#### 3.3.5. Psychosocial

The validated Malay version geriatric depression scale (GDS-15) was used to assess potential depressive symptoms among the participants [35], whereas the disability status was measured using the 12-item version of WHODAS 2.0 [36].

#### 3.3.6. Dietary Intake

The dietary intake was obtained using the Dietary History Questionnaire (DHQ) [37]. The nutrient intake was analyzed using the Nutritionist Pro software. The output from the software was then exported into the Excel database.

#### 3.3.7. Biochemical Analysis

A total of 20 ml fasting peripheral venous blood was withdrawn using a butterfly syringe by a phlebotomist for the biochemical analysis and included fasting blood sugar, HbA1c, total cholesterol, high-density lipoprotein (HDL), low-density lipoprotein (LDL), triglyceride, and albumin.

### 3.4. Statistical Analysis

The cumulative incidence for CF was calculated using the number of new cases of CF during the follow-up period divided by the number of participants at risk in the population at the beginning of the study. The CF incidence rate was calculated as the number of new cases of CF divided by the total person-time observed between the two assessments. The age-specific incidence rate of CF was computed for 5-year age categories (60–64, 65–69, 70–74, and 75 years and above) using a person-years analysis. Participants were considered at risk and contributed to the person-years from the date of their baseline evaluation to the date of the follow-up where they were determined to have CF or not. The age-specific incidence rate curve was modeled using the Microsoft Excel database. 

Descriptive statistics were used to present characteristics of participants according to the dropout or non-dropout and presence or absence of CF. For the dropout analysis, the dependent variable was the dropout as the reference variable, compared to non-dropout participants. The data of participants who had died within these 5 years and who had CF at the baseline were excluded from the dropout analysis to identify the significant demographic factors that predicted dropout among our participants. Meanwhile, for the analysis on the predictors of CF, the dependent variable was the CF group as the reference variable, compared to the non-CF group. The reported findings were factors from the baseline and not from the follow-up data. The sociodemographic factors, blood pressure, anthropometric, body composition, physical fitness test, cognitive assessments, biochemical indices, and dietary intake were compared between these two groups using a Chi-Square test (ꭕ²) for categorical variables and independent t-test for continuous variables. As for the smoking and alcohol drinking data, both current and former participants were classified as smokers and alcoholic drinker groups. The results were reported as n (%) and the mean ± standard deviation for normally distributed data. The significant value was set at *p* < 0.01. 

A hierarchical binary logistic regression (BLR) was performed to determine the predictors of CF incidence in a multivariate model. For the first stage, all the significant variables (*p* < 0.01) from the univariate analysis were adjusted for multiple testing and classified into five different groups, as follows: (1) sociodemographic and medical status; (2) blood pressure, anthropometry, clinical profile, and biochemical indices; (3) social support, functional and depression status; (4) fitness and cognitive assessments; and (5) dietary intake associated with cognitive frailty. All covariates (age, sex, and total years of education) were included as control variables in each model. Then, all significant variables (p < 0.01) from each model were included in the final forward-stepwise logistic model. The physical frailty and mild cognitive impairment were also included in the final model by controlling all the confounding variables. The significant variables that appeared in the final model were the predictors of CF among participants (*p* < 0.05). The final model fit was evaluated using the Receiver Operating Characteristic Curve (ROC), specificity, sensitivity, and the area under the curve (AUC) with 95% confidence intervals (CIs). These analyses were performed using SPSS version 23.0 (Licensed materials - Property of SPSS Incorporation an IBM Company Copyright 1989 and 2010 SPSS, Chicago, United States).

## 3. Results

**Figure 2:**
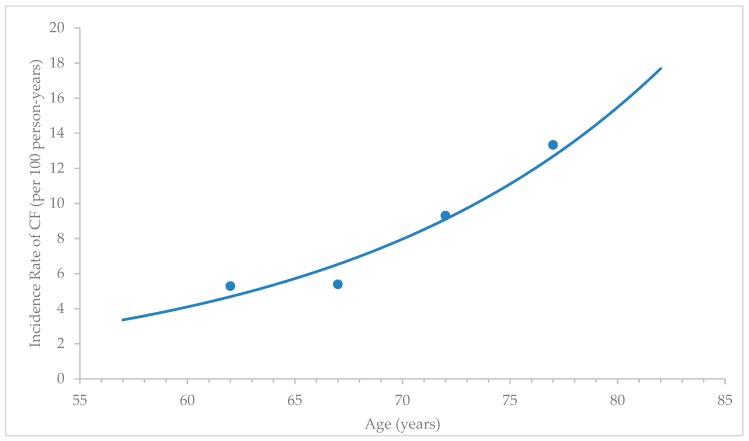
The age-specific incidence rates and 95% confidence intervals of cognitive frailty events from 2013 to 2018. The incidence rate was computed for 5 age categories using a person-years analysis and was plotted at the average age for each age category: 62 years for the 60-64 years category, 67 years for the 65–69 category, 72 years for the 70-74 years category, and 77 years for 75 years and above. Note: CF = cognitive frailty.

Out of 490 participants without CF at the baseline, 282 (70.5%) participants were successfully followed up after 5 years (Response rate 57.6%). In the present study, 182 (64.5%) participants remained without CF, and 100 (35.5%) developed CF. In this group, the 5 years’ cumulative incidence of cognitive frailty was 35.5%. The observed incidence rate of CF within the 5-years period had a value of 7.1 per 100 person-years. Figure 2 shows the estimated age-specific incidence rate of CF from the baseline to the 5-years follow-up. Rates increased with age from 5.28 per 100 person-years (95% CI: 3.33–7.24) in the 60-64 years age group, to 5.38 per 100 person-years (95% CI: 3.08–7.69) in the 65–69 years age group, to 9.50 per 100 person-years (95% CI: 6.12–12.47) in the 70-74 years age group, to 13.34 per 100 person-years (95% CI: 7.17–19.49) in the 75 years and above age group. Based on this projection, the incidence rate of CF was expected to increase by two-fold with every 10 year-increase in age. 

The baseline characteristics, cognitive and physical status of the dropout (N = 169), and the non-dropout groups (N = 282) are presented in Table 1. The participants in the dropout group were significantly older and living alone at the baseline compared to those who did not drop out (*p* < 0.01). The analysis was further analyzed in the binary logistic regression, where only living alone (OR = 2.60, 95% CI: 1.42–4.75, *p* < 0.05) was significantly associated with dropping out. There is no significant difference in the physical and cognitive status between the dropout and non-dropout groups (*p* > 0.05). 

Table 2 presents the baseline characteristics for the participants who were not in the CF group and for those who developed CF. Participants with CF were found to be older, living alone, having lower mean years of education, and having a lower household income compared to those without CF (*p* < 0.01). 

Compared to the non-CF participants, the body fat percentage and systolic blood pressure were significantly higher, whereas the fat-free mass, skeletal muscle mass, and calf circumference were lower among participants with CF (*p* < 0.01). Participants with CF had a lower physical fitness (2-minute step, chair stand test, TUG, and back scratch tests) and cognitive assessments (MMSE, MoCA, Digit span, RAVLT, Digit symbol, VR I, and VR II) compared to those who were non-CF (*p* < 0.01 for all parameters). Participants with CF had a higher score of GDS and WHODAS and lower ADL and IADL, indicating higher levels of depression and disability in comparison to older adults who were non-CF (*p* < 0.01 for all parameters). 

With regards to the dietary intake (Table 3), the energy and macronutrient intake (carbohydrate and fat) appeared to be lower among participants with CF compared to those who were non-CF; however, the differences were not significant (*p* > 0.01). Meanwhile, total fiber, vitamin C, vitamin D, alpha-tocopherol, folate, calcium, copper, and magnesium were significantly lower among participants with CF as compared to those who were non-CF (*p* < 0.01 for all parameters). 

In Table 4, the results from the hierarchical logistic regression indicated that advancing age (OR = 1.12, 95% CI:1.044 – 1.208, *p* < 0.05), a higher GDS score (OR = 1.200, 95% CI:1.051 – 1.369, *p* < 0.05), lower digit symbol score (OR = 0.668, 95% CI:0.556 – 0.803, *p* < 0.05), longer time to complete the TUG test (OR = 1.232, 95% CI:1.039 – 1.460, *p* < 0.05), low intake of vitamin D (OR = 0.362, 95% CI: 0.141 – 0.930, *p* < 0.05), and physical frailty (OR = 2.157, 95% CI: 1.016 – 4.580), *p* < 0.05) were found to be a predictors of CF in this study. The final model was a good fit for the data, with 77.6% of the variability, which was satisfactory to predict the cognitive frailty incidence. The ROC curves with the area under the curve score (AUC = 82.6%) showed the accuracy of the final model with a good sensitivity (81.1%) and specificity (76.1%) for predicting CF among older adults. 

## 4. Discussion

This study has successfully reported the incidence rate of CF at a rate of 7.1 per 100 person-years among older adults who did not exhibit CF at the baseline. The incidence rate of mild cognitive impairment (MCI) and physical frailty was 10.5 and 6.8 per 100 person-years, as reported in previous local studies [38,39]. Accordingly, the incidence rate of CF from the recent study was relevant and proportional to the reported incidence rate for both geriatric syndromes. Apart from that, the incidence rate increased with age and doubled approximately every 10 years, with estimates as high as 13.34 per 100 person-years for older adults aged 75 years and above. The separation of older participants into smaller age categories allows for more specific incidences rate and trajectories [40]. Shimada et al. [41] reported that individuals with CF had a higher risk of dementia than healthy older adults or older adults with either physical frailty or cognitive impairment alone. By recognizing the consequences of CF on the increased incidence of dementia [5], secondary preventive strategies should be warranted, such as multidomain interventions that target physical, nutritional, cognitive, and psychological factors to delay the progression toward dementia and adverse health-related outcomes, particularly disability, hospitalization, and mortality. 

Advancing age and depression were reported as predictors of the occurrence of CF in the present longitudinal analyses and in our previous cross-sectional analyses [18]. We believe that the other non-longitudinal factors previously reported [18], including the lack of social support, low functional status, and niacin deficiency did not cause an effect on the occurrence of CF among our local population. For example, CF may lead to social isolation, poor physical function [42], and a nutriment deficit [43]. Our study results did not demonstrate functional limitation and disability as the predictors of CF incidence, as they are considered to be the consequences of CF rather than the predictors [44,45]. Furthermore, chronic diseases may exacerbate functional decline and cognitive impairment [46]. Moreover, not all diseases are able to predict CF [47,48]. Hence, our study findings may suggest that the development of CF may not be caused by the mechanisms that are shared with chronic diseases. As stated earlier, although in a few studies factors associated with CF have been reported [18,49], this came from cross-sectional observations which do not provide definite information about the cause-and-effect relationships, as compared to longitudinal study reports. 

Our current study results highlighted the fact that both advancing age and depression have a major role in the development of CF. Aging is an established predominant risk factor for both frailty and cognitive impairment [50,51] and appears to be further exacerbated by the presence of depression [52]. The majority of Asian older adults are reported to have later-life depression issues and are more often under-treated due to the misconception that this is part of the aging process [53]. The co-existence of untreated depression with physical frailty poses increased risks of negative consequences in older age, such as a lower quality of life, increased morbidity, disability, and accelerated cognitive impairment [54], which eventually cause dementia. Hence, the early detection of depressive symptoms in older adults may help to prevent the occurrence of CF and enhance the health-related quality of life in this growing population. 

Simple physical performance assessment tools, such as the timed-up-and-go test (TUG) and gait speed tests have been used as markers of the frailty phenotype among community-dwelling older adults [55]. Our study findings demonstrated that a one-unit increase in the TUG test significantly increased the odds of developing CF among an older population. TUG is a well-known test for examining functional mobility and is a simple marker of gait impairments that correlate with cognitive impairment and dementia [56,57]. In addition, executive function and processing speed were demonstrated to be associated with gait variability and turning in the TUG task [58,59]. Furthermore, recent study findings showed that a one-unit decrease in the information processing speed test, as assessed using a digit symbol score, increased the incidence of CF by 33.2%. The digit symbol task has been shown to be a sensitive method for detecting the decrements in cognitive function, specifically in the processing speed among older people with DM and depression [60,61]. Moreover, the association of a smaller volume of the pre-frontal area with a slow gait speed was explained by the lower performance in the digit symbol test [9]. It is interesting that the use of a single cognitive domain could predict the development of CF better than the global cognitive test could. This is probably because specific cognitive domains such as processing speed, executive function, and attention have been shown to be associated with frailty indicators like gait speed and grip strength [62]. In addition, the processing speed was found to partly mediate and attenuate the association of walking speed with the incidence of dementia, as compared to global cognition [10]. It is possible that not all cognitive domains may be impaired simultaneously in older adults with CF, but that they are specifically dependent on the age and frailty indicators present [62]. Although findings from our study were not able to clarify whether cognitive deficits mediated gait and balance dysfunction or vice versa, they demonstrated that both tests are able to forecast the incidence of both physical frailty and cognitive impairment. Therefore, we suggest that both TUG and digit symbol task tests could be the best simple screening tools for predicting the incidence of CF among older adults. 

Our study results also demonstrated that an inadequate intake of vitamin D could also predict the incidence of CF. It is noteworthy that participants categorized as CF groups had a low intake of vitamin D, which did not even meet the requirement suggested for older adults (20 µg/day) [63]. Inadequate vitamin D intake has a negative implication on health, including psychological impacts such as mood disorder and depression [64] that could lead to the development of CF among an older population. The established relationship between a low vitamin D intake and frailty can be explained using the two biological pathways related to muscle and bone health [65]. Additionally, the well-known consequence of low vitamin D in calcium absorption and bone mineralization [65] is associated with a reduction in muscle mass and an impaired muscle function [66,67]. As a consequence, there are increased risks of falls and fractures among older adults. Moreover, an inadequate vitamin D intake has been reported to significantly increase the risk of MCI related to domain-specific cognitive performance, particularly executive function and information processing speed [68,69]. Therefore, the insufficiency of vitamin D intake may have some synergistic effect on both the underlying mechanisms of cognitive impairment and on physical frailty, as a result increasing the risk of poor clinical outcomes in older adults with cognitive frailty. 

Our study results indicate that physical frailty predicts CF better than MCI does. This concurs with previous reports showing the directional relationship of cognitive impairment, which was caused by physical impairments and not by the presence of concomitant neurological impairments [8,70]. In a longitudinal study, frail individuals with normal cognition were detected to have cognitive impairment after a 5-years follow-up and were associated with a greater risk of developing dementia of the AD type [71]. The proposed terminology of CF may represent a state of reduced brain neurophysiological reserves (brain frailty), a condition where both neurodegenerative and vascular diseases as well as physical frailty present concomitantly [8,72]. Hence, an early identification of older adults with physical frailty may represent a step forward in formulating the strategies to prevent the development of CF, which could further aggravate the occurrence of dementia and disability [5,19]. 

There are several strengths in the current study. First, this is the first report on the incidence rate of CF among older adults in Malaysia using longitudinal data. Second, this study involved a wide range of parameters with a detailed protocol covering several domains such as fitness, cognitive function, nutrient intake, anthropometric measurements, body composition, psychosocial function, and biochemical indices as predictors of CF. Third, this longitudinal study at a five-years endpoint can be considered as a fairly long period for incidence rate analyses with strong predictors of CF. Nevertheless, there are several limitations to this study. It should be noted that this study only involved two out of 14 states in Malaysia, with a smaller sample size at the follow-up. This is inevitable when involving older adults. Furthermore, the participants at the follow-up were mostly Chinese, followed by Malay and Indians, which does not match the national composition of the Malaysian population. The drop-out rate was fairly high, thus suggesting that these participants were more likely to be older and living alone. Older adults living alone have been identified as a vulnerable risk group with a higher risk of CF [18]. This group is often associated with having difficult living situations, limited resources, and a lack of support for acquiring care, medication management, and transportation [73]. Therefore, it is possible that such issues could have restricted participation in the follow-up sessions, potentially leading to a higher dropout rate among our participants. Alternative methods such as home visits to capture the data of these participants could have reduced dropouts and improved longitudinal follow-up rates. Notably, the percentage of those who died was also high, and hence some of these participants might have developed CF during the 5-year period and were missed out from the incidence analysis. This could have led to a possible underestimation of CF incidence rate in our study. There is also a need to measure the impact of CF on the mortality rate. Lastly, we would recommend to conduct inflammatory, oxidative stress biomarkers and metabolomic approach analysis to identify the specific biomarkers or metabolites underlying the CF development. 

## 5. Conclusions

The incidence rates of CF and dementia among Malaysian older adults were 7 per 100 per-years and 1.25 per 100 person-years at a five years follow-up. Advancing age, depression, slow processing speed, lower performance in the TUG test, low intake of vitamin D, and physical frailty were predictors in the development of CF among Malaysian older adults. Future investigation into the relationship between CF and dementia and its related pathologies are needed to construct targeted prevention and intervention strategies to decelerate the development of CF among older adults.

## Figures and Tables

**Table 1:** The baseline characteristic, physical and cognitive status of dropout and non-dropout participants [presented as mean ± standard deviation (sd) or n (%)].

Parameters	N = 694		*p*-Value
	Dropout (n = 169)	Non-Dropout (n = 282)	
Age, mean ± sd	68.19 ± 5.79	67.00 ± 4.98	0.020
Gender:			
Male	77 (45.8%)	126 (44.7%)	0.707
Female	92 (54.2%)	156 (55.3%)	
Ethnic:			
Malay	60 (35.5%)	120 (43.3%)	0.056
Chinese & Indian	109 (64.5%)	162 (56.7%)	
Education years, mean ± sd	6.51 ± 4.51	6.98 ± 4.64	0.284
Household income, mean ± sd	1806.39 ± 2579.66	1945.88 ± 2814.25	0.585
Social support, mean ± sd	40.29 ± 13.45	39.07 ± 14.54	0.363
Living alone	38 (22.4%)	18 (6.4%)	0.001 *
Smoking	46 (27.2%)	50 (17.7%)	0.655
Alcohol drinking	16 (9.5%)	16 (5.7%)	0.855
Diseases:			
Diabetes	64 (37.9%)	53 (18.8%)	0.067
Hypertension	111 (65.7%)	110 (39.0%)	0.190
Hypercholesterolemia	62 (36.7%)	68 (24.1%)	0.917
Heart disease	17 (10.1%)	12 (4.26%)	0.249
Mild Cognitive Impairment (MCI)	44 (26.0%)	53 (20.6%)	0.337
Frailty status (pre-frailty & frailty)	134 (79.3%)	139 (49.3%)	0.293

* Significant at *p* < 0.01; Notes: CF = cognitive frailty.

**Table 2:** The baseline attributes of the participants detected with CF and non-CF at the 5-years follow-up [presented as mean ± standard deviation (sd) or n (%)].

Baseline Parameters	CF Status at 5 Years		
	N = 282		*p*-Value
	CF (n = 100)	Non-CF (n = 182)	
Age, mean ± sd:	69.09 ± 5.70	66.25 ± 4.54	<0.001 *
Sex			
Men	37 (37.0%)	89 (48.9%)	0.061
Women	63 (60.3%)	93 (51.1%)	
Ethnic			
Malay	47 (47.0%)	74 (40.7%)	0.317
Chinese & Indian	53 (53.0%)	108 (59.3%)	
Education (years), mean (sd):	3.67 ± 3.33	8.08 ± 4.50	<0.001 *
Living alone	14 (14.0%)	10 (5.5%)	0.024
Smoker	18 (18.0%)	38 (20.9%)	0.641
Alcohol drinking	4 (4.0%)	13 (7.1%)	0.433
Household income, mean ± sd	1172.73 ± 1511.22	2108.97 ± 305.90	0.001 *
Diseases			
Diabetes	29 (29.0%)	34 (18.7%)	0.053
Hypertension	53 (53.0%)	75 (41.2%)	0.062
Hypercholesterolemia	31 (31.0%)	48 (26.4%)	0.410
Heart Disease	9 (9.0%)	7 (3.8%)	0.104
Body Mass Index, mean ± sd	25.88 ± 4.19	25.24 ± 3.98	0.210
% Body Fat, mean ± sd	40.69 ± 10.70	37.15 ± 9.78	0.005 *
Fat mass (kg), mean ± sd	25.33 ± 8.58	24.25 ± 8.71	0.316
Fat free mass (kg), mean ± sd	36.34 ± 7.85	39.95 ± 8.10	<0.001 *
Skeletal muscle mass (kg), mean ± sd	19.26 ± 4.68	21.48 ± 4.85	<0.001 *
Fasting blood sugar (mmol/l), mean ± sd	6.28 ± 2.64	5.95 ± 2.01	0.287
HbA1c (%), mean ± sd	15.01 ± 2.85	14.45 ± 2.55	0.124
Total cholesterol (mmol/l), mean ± sd	5.35 ± 1.08	5.11 ± 0.99	0.091
High density lipoprotein (mmol/l), mean ± sd	1.46 ± 0.37	1.44 ± 0.38	0.740
Low density lipoprotein (mmol/l), mean ± sd	3.18 ± 0.97	3.06 ± 0.93	0.338
Triglyceride (mmol/l), mean ± sd	1.60 ± 0.73	1.39 ± 0.75	0.032
Albumin (g/l), mean ± sd	43.21 ± 2.93	42.91 ± 2.86	0.452
Blood Pressure:			
Diastolic (mmHg), mean ± sd	77.31 ± 10.39	76.32 ± 19.40	0.542
Systolic (mmHg), mean ± sd	143.09 ± 17.73	136.02 ± 19.40	0.003 *
Calf Circumference (cm), mean ± sd	33.88 ± 3.16	34.67 ± 3.53	0.062
Waist Circumference (cm), mean ± sd	89.67 ± 10.27	88.81 ± 10.55	0.512
Hip Circumference (cm), mean ± sd	97.94 ± 8.88	98.39 ± 8.61	0.677
MUAC (cm), mean ± sd	29.10 ± 3.25	29.08 ± 3.05	0.965
Psychosocial and functional status:			
GDS, mean ± sd	2.57 ± 2.05	1.92 ± 2.06	<0.001 *
ADL, mean ± sd	37.16 ± 9.31	43.91 ± 10.11	<0.001 *
IADL, mean ± sd	12.61 ± 2.08	13.48 ± 1.10	<0.001 *
WHODAS, mean ± sd	7.49 ± 7.86	3.55 ± 6.17	<0.001 *
Neurocognitive			
Mild Cognitive Impairment (MCI)	42 (42.4%)	51 (28.0%)	0.017
PCA of cognitive test	-0.31 ± 0.90	0.78 ± 0.94	<0.001 *
MMSE, mean ± sd	21.90 ± 4.99	25.79 ± 3.47	<0.001 *
MoCA, mean ± sd	17.88 ± 5.03	22.25 ± 4.77	<0.001 *
Span Digit, mean ± sd	7.29 ± 2.50	8.51 ± 2.28	<0.001 *
RAVLT, mean ± sd	36.71 ± 8.83	43.14 ± 10.64	<0.001 *
Digit symbol, mean ± sd	4.23 ± 1.70	7.28 ± 3.32	<0.001 *
VR I, mean ± sd	38.37 ± 29.92	59.64 ± 33.33	<0.001 *
VR II, mean ± sd	29.23 ± 32.27	53.41 ± 27.55	<0.001 *
Frailty status (pre-frail & frailty)	100 (100.0%)	136 (74.7%)	<0.001 *
Fitness test			
2-minute step test, mean ± sd	62.50 ± 25.81	73.57 ± 23.86	<0.001 *
Chair stand test, mean ± sd	9.69 ± 2.78	11.56 ± 2.84	<0.001 *
Chair sit and reach test, mean ± sd	1.34 ± 9.62	1.16 ± 9.09	0.876
TUG test, mean ± sd	10.91 ± 2.90	8.62 ± 1.99	<0.001 *
Back scratch test, mean ± sd	17.51 ± 12.28	9.98 ± 10.98	<0.001 *

* Significant at *p* < 0.01. Notes: CF = cognitive frailty; MUAC = Mid-upper arm circumferences; ADL = activities of daily living; IADL = instrumental activities of daily living; GDS = Geriatric Depression Scale; WHODAS = WHO Disability Assessment Schedule; MOSS = Medical Outcome Study Social Support Survey; MMSE = Mini-Mental State Examination; MoCA = Montreal Cognitive Assessments; RAVLT = Rey Auditory Verbal Learning Test; VR I = visual reproduction I; VR II = visual reproduction II; TUG = Timed-up-and-Go test; and sd = standard deviation.

**Table 3:** The dietary intake among participants at the baseline detected with CF and non-CF at the 5-years follow-up [presented as mean ± standard deviation (sd) or n (%)].

Nutrient Intake at Baseline	CF Status at 5 Years		
	N = 282		*p*-Value
	CF (n = 100)	Non-CF (n = 182)	
Energy (kcal)	1587 ± 411	1679 ± 406	0.080
Protein (g/day)	68.90 ± 21.14	70.70 ± 20.41	0.498
Protein (% of energy)	17.62 ± 3.96	17.04 ± 3.40	0.208
Carbohydrate (g/day)	207.22 ± 68.76	224.42 ± 67.90	0.050
Carbohydrate (% of energy)	51.65 ± 8.06	53.26 ± 8.28	0.127
Fat (g/day)	53.57 ± 16.67	54.41 ± 17.66	0.704
Fat (% of energy)	30.70 ± 7.18	29.28 ± 6.98	0.117
Total Fibre (g/day)	4.10 ± 2.42	5.04 ± 2.62	0.004 *
Vitamin C (mg/day)	109.57 ± 75.51	145.86 ± 90.07	0.001 *
Vitamin D (mg/day)	0.11 ± 0.32	0.49 ± 1.02	<0.001 *
Vitamin E (mg/day)	6.68 ± 19.16	5.52 ± 3.26	0.443
Vitamin K (mg/day)	19.39 ± 87.73	34.85 ± 91.24	0.181
Alpha-Tocopherol (mg/day)	0.26 ± 0.78	0.78 ± 1.34	<0.001 *
Thiamin (mg/day)	1.53 ± 3.20	1.61 ± 3.32	0.844
Riboflavin (mg/day)	1.18 ± 0.50	1.33± 0.44	0.010
Niacin (mg/day)	9.83 ± 3.35	10.81 ± 4.32	0.054
Pyridoxine (mg/day)	0.70 ± 0.32	0.76 ± 0.36	0.229
Panthotenic acid (mg/day)	0.23 ± 0.41	0.37 ± 0.47	0.011
Folate (µg /day)	93.43 ± 58.39	119.26 ± 68.38	0.002 *
Calcium (mg/day)	439.35 ± 151.82	540.36 ± 222.90	<0.001 *
Sodium (mg/day)	1602.59 ± 992.36	1362.28 ± 710.87	0.039
Iron (mg/day)	12.98 ± 4.73	14.14 ± 4.63	0.052
Zinc (mg/day)	3.44 ± 1.78	3.87 ± 1.74	0.055
Copper (mg/day)	0.57 ± 0.33	0.71 ± 0.39	0.004 *
Magnesium (mg/day)	126.32 ± 62.81	149.32 ± 66.98	0.006 *
Selenium (µg/day)	25.13 ± 22.54	28.07 ± 17.71	0.242

* Significant at *p* < 0.01 using Independent t-test; Notes: sd = standard deviation.

**Table 4:** Potential predictors for cognitive frailty at 5 years (N = 282).

Predictor of Interest	B	ORAdj (95% CI)	*p*-Value
Age (years)	0.116	1.123 (1.044–1.208)	0.002 *
Digit symbol (scale score, continuous)	−0.403	0.668 (0.556–0.803)	<0.001 *
TUG test (continuous)	0.209	1.232 (1.039–1.460)	0.016 *
GDS (total score, continuous)	0.182	1.200 (1.051–1.369)	0.007 *
Vitamin D (µg, continuous)	−1.016	0.362 (0.141–0.930)	0.035 *
Physical frailty (yes vs. no)	0.769	2.157 (1.016–4.580)	0.045 *

* Significant at *p* < 0.05 using forward-stepwise Binary Logistic Regression. Notes: GDS = Geriatric Depression Scale; WHODAS = WHO Disability Assessment Schedule; TUG = Timed-up-and-Go test; and sd = standard deviation.
